# Monitoring the Zero-Inflated Time Series Model of Counts with Random Coefficient

**DOI:** 10.3390/e23030372

**Published:** 2021-03-20

**Authors:** Cong Li, Shuai Cui, Dehui Wang

**Affiliations:** 1 School of Mathematics, Jilin University, Changchun 130012, China; li_cong@jlu.edu.cn (C.L.); cuishuai@jlu.edu.cn (S.C.); 2 School of Economics, Liaoning University, Shenyang 110036, China

**Keywords:** CUSUM control chart, INAR-type time series, statistical process monitoring, random survival rate, zero-inflation

## Abstract

In this research, we consider monitoring mean and correlation changes from zero-inflated autocorrelated count data based on the integer-valued time series model with random survival rate. A cumulative sum control chart is constructed due to its efficiency, the corresponding calculation methods of average run length and the standard deviation of the run length are given. Practical guidelines concerning the chart design are investigated. Extensive computations based on designs of experiments are conducted to illustrate the validity of the proposed method. Comparisons with the conventional control charting procedure are also provided. The analysis of the monthly number of drug crimes in the city of Pittsburgh is displayed to illustrate our current method of process monitoring.

## 1. Introduction

This work is motivated by an empirical analysis and process control of a monthly drug crime series, which contains excess zeros (over 40%) and shows clear serial dependence (see Section 5 for more details). To solve this problem, an appropriate integer-valued model is selected, further, control charts based on this model are developed. Among kinds of integer-valued models, a specific kind featured with first-order integer-valued autoregressive (INAR(1)) models plays an very important role and has been widely studied in the literature. In reality, serial dependence among the count data have been demonstrated to arise extensively in practice, typical examples are infectious disease counts, defect counts and unemployment counts, etc. These data are important indicators of the epidemic study, quality control and economics analysis, and the process monitoring is essential to detect the shifts in them.

The first INAR(1) model proposed by Al-Osh and Alzaid [1] is in the following form

$$X_{t}=\alpha \circ X_{t-1}+\varepsilon_{t},\;\;\;\;t=1,2,\cdots ,$$

where the binomial thinning operator “$\circ^{\prime \prime }$ is defined by Steutel and Van Harn [2], $\alpha \circ X_{t-1}=\sum_{i=1}^{X_{t-1}}Y_{i}$, α∈(0,1), ${\left\{Y_{i}\right\}}_{N}$ is a sequence of independent and identically distributed (i.i.d.) random variables with Bernoulli(α) distribution; and ${\left\{\varepsilon_{t}\right\}}_{N}$ is a sequence of i.i.d. random variables, independent of all $\left\{Y_{i}\right\}$. The INAR(1) model is currently applied in various kinds of real-world problems because of its good interpretability. As one example, we let $X_{t}$ represent the number of patients of an infectious disease in a community at time *t*, $\varepsilon_{t}$ the number of new patients entering the community at time *t*, and suppose each patient at time t−1 survives at time *t* with survival probability α. As for the crime data, $\alpha \circ X_{t-1}$ can be considered as the number of re-offendings provoked by $X_{t-1}$ with probability α. Depending on the nature of this kind of observed data, the INAR(1) models have been modified and generalized with respect to their orders (Ristić and Nastić [3], Nastić, Laketa and Ristić [4]), dimensions (Pedeli and Karlis [5], Khan, Cekim and Ozel [6]), marginal distributions (Alzaid and Al-Osh [7], Alzaid and Al-Osh [8], Jazi, Jones and Lai [9], Ristić, Nastić and Bakouch [10], Barreto-Souza [11]), thinning operators (Ristić, Bakouch and Nastić[12], Liu and Zhu [13]), and mixed models (Ristić and Nastić [3], Li, Wang and Zhang [14], Orozco, Sales, Fernández and Pinho [15]). For more literature, we refer to review papers (Weiß [16], Scotto, Weiß and Gouveia [17]). Differing with the models that based on a fixed survival rate α, Zheng, Basawa and Datta [18] proposed the random coefficient INAR(1) model, supposing that the parameter α may be affected by various environmental factors, and could vary randomly over time. Some of the generalizations of random coefficient INAR models can also be found in Kang and Lee [19] and Zhang, Wang and Zhu [20]. In particular, considering both random survival probability and zero-inflation phenomenon, Bakouch, Mohammadpour and Shirozhan [21] purposed a zero-inflated geometric INAR(1) time series with random coefficient (short for the ZIGINAR${}_{\mathrm{RC}}$(1) process). The ZIGINAR${}_{\mathrm{RC}}$(1) model has simple structure and good properties, which turns out to be the best fit for the real data studied by us.

As the serial dependence shows big influence on the performance of the control chart, the traditional control charts under the assumption of independent observations are not appropriate in many cases. Therefore, the monitoring of INAR(1) models has received much attention. The related research includes but not limited to the control charts for the generally developed Poisson INAR(1) models (Weiß [22], Weiß and Testik [23], Weiß and Testik [24], Yontay, Weiß, Testik and Bayindir [25]), for zero-inflated or zero-deflated INAR(1) models (Rakitzis, Weiß and Castagliola [26], Li, Wang and Sun [27], Fernandes, Bourguignon and Ho [28]), for the mixed INAR(1) model (Sales, Pinho, Vivacqua and Ho [29]), etc. While, to the best of our knowledge, methods for monitoring the zero-inflated INAR(1) model with random coefficient have not been studied in the literature so far, which is exactly what we are going to explore. As cumulative sum (CUSUM) control charts are known to be sensitive in detecting small shifts, we study the performance of the CUSUM chart for monitoring ZIGINAR${}_{\mathrm{RC}}$(1) process. We investigate the practical guidelines for the statistical design and the methods for evaluating the chart performance. Besides monitoring mean shifts of the ZIGINAR${}_{\mathrm{RC}}$(1) model, our scope is also to monitor correlation shifts in the model. Meanwhile, we compare the performance of the CUSUM chart with the conventional Shewhart chart.

The rest of the article is outlined as follows. The ZIGINAR${}_{\mathrm{RC}}$(1) process and some properties of this process are introduced in Section 2. In Section 3, we present the monitoring procedure to detect the mean and correlation shifts of the process. Extensive computation results are discussed in Section 4. In Section 5, the applicability of the process monitoring is investigated using the monthly number of drug crimes in Pittsburgh. Finally conclusions and possible future lines of research are shown in Section 6.

## 2. The ZIGINAR${}_{\mathrm{RC}}$(1) Process

A randomized binomial thinning operation in Bakouch, Mohammadpour and Shirozhan [21] is defined by

$$\alpha_{t}\circ X=\left\{\begin{array}{cc} \alpha \circ X, & \mathrm{with}\;\mathrm{probability}\;1-\beta ,\\ 0, & \mathrm{with}\;\mathrm{probability}\;\beta , \end{array}\right.$$

where α,β∈(0,1), $\alpha_{t}$ is a binary random variable independent of discrete random variable *X*, $P\left(\alpha_{t}=0\right)=\beta =1-P\left(\alpha_{t}=\alpha \right)$.

Based on the definition of the randomized binomial thinning operation, the ZIGINAR${}_{\mathrm{RC}}\left(1\right)$ model $\left\{X_{t}\right\}$ presented by Bakouch, Mohammadpour and Shirozhan [21], is given by

$$X_{t}=\alpha_{t}\circ X_{t-1}+\varepsilon_{t},\;\;\;\;t=1,\;2,\cdots ,$$

where the marginal distribution is a zero-inflated Geometric distribution (denoted as ZIG(p,θ)), $P\left(X_{t}=0\right)=p+\left(1-p\right)/\left(1+\theta \right)$, $P\left(X_{t}=j\right)=\left(1-p\right)\theta^{j}/{\left(1+\theta \right)}^{j+1},\;j=1,2,\cdots ,$ and p∈(0,1),θ>0. ${\left\{\varepsilon_{t}\right\}}_{N}$ is independent of the past of the solution $\left\{X_{s};s<t\right\}$ and the binary sequence $\left\{\alpha_{t}\right\}$, parameters are also constrained by the condition p/(β+p(1−β))<α<1.

The ZIGINAR${}_{\mathrm{RC}}$(1) process is quite suitable for modelling some real-life phenomena in which counted events may survive or vanish with the random survival probability $\alpha_{t}$. Such series are studied in Section 5 with an example of the counts of the drug crimes, where the re-offending rate may be affected by public security situation and financial situation. The mean, variance, and first-order autocorrelation function of the process are, respectively,

$$\mu_{X}≜E\left(X_{t}\right)=\left(1-p\right)\theta ,\;\sigma_{X}^{2}≜\mathrm{Var}\left(X_{t}\right)=\left(1-p\right)\theta \left[\left(1+p\right)\theta +1\right],$$

$$\rho_{X}≜Corr\left(X_{t},X_{t+1}\right)=\alpha \left(1-\beta \right).$$

**Figure 1:**
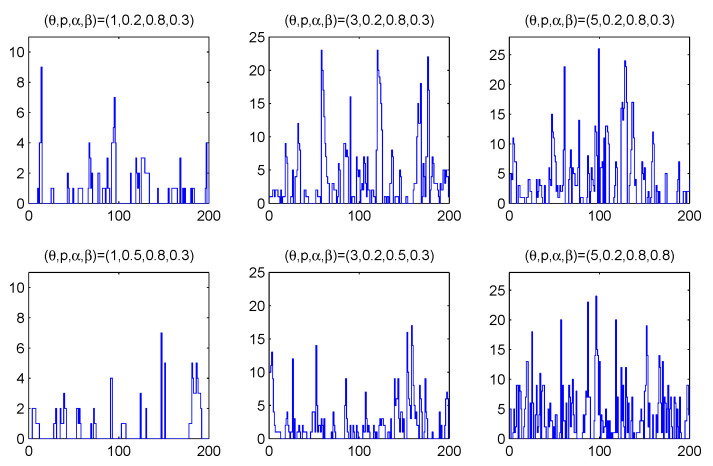
Sample path of ZIGINAR${}_{\mathrm{RC}}$(1) processes for θ=1,3,5, p=0.2,0.5, α=0.5,0.8 and β=0.3,0.8.

Obviously the process is characterized by the property of overdispersion, i.e., the variance greater than the expectation. Figure 1 shows some sample paths of simulated ZIGINAR${}_{\mathrm{RC}}$(1) processes for θ=1,3,5; p=0.2,0.5; α=0.5,0.8 and β=0.3,0.8. As we can see, the model has larger process mean with larger θ, and larger percentage of zeros with larger *p*.

Following Theorem 2.1 in Bakouch, Mohammadpour and Shirozhan [21], the ZIGINAR${}_{\mathrm{RC}}\left(1\right)$ model has a unique, strictly stationary solution given by

$$\sum_{i=1}^{\infty }\left(\prod_{l=0}^{i-1}\alpha_{t-l}\right)\circ \varepsilon_{t-i}+\varepsilon_{t}.$$

Furthermore, the probability mass function of ${\left\{\varepsilon_{t}\right\}}_{N}$ is

$$\begin{array}{cc} P(\varepsilon_{t}=j)= & \frac{p}{\beta +p(1-\beta )}I_{\left(j\right)}+\frac{\left(1-p)(1-\alpha \right)}{1-\alpha [\beta +p(1-\beta )]}\frac{\theta^{j}}{{\left(1+\theta \right)}^{j+1}}\\ \; & +\frac{\left(1-p)(1-\beta )[\alpha (\beta +p(1-\beta ))-p\right]}{\left(1-\alpha [\beta +p(1-\beta )])(\beta +p(1-\beta )\right)}\\ \; & \times \frac{{\left[\alpha \theta \left(\beta +p\left(1-\beta \right)\right)\right]}^{j}}{{\left[1+\alpha \theta \left(\beta +p\left(1-\beta \right)\right)\right]}^{j+1}},\;\;\;\;\;\;\;\;\;j=0,1,2,\cdots . \end{array}$$

where $I_{\left(j\right)}=1$ for j=0 and 0 else. It can be deduced that the innovation series ${\left\{\varepsilon_{t}\right\}}_{N}$ is a mixture of three random variables, a degenerate distribution at 0, Geometric(θ/(1+θ)) and Geometric(αθ(β+p(1−β))/(1+αθ(β+p(1−β)))) distributions with three different mixing portions. The following form is the transition probability of the process ${\left\{X_{t}\right\}}_{N_{0}}$

P(Xt=j|Xt−1=i)=βP(εt=j)+(1−β)∑l=0min(i,j)ilαl(1−α)i−lP(εt=j−l),i,j=0,1,⋯.

Some other important probabilistic properties of the process, like spectral density, multi-step conditional mean and variance, extreme order statistics, distributional properties of length of run of zeros, have also been discussed in Bakouch, Mohammadpour and Shirozhan [21]. Furthermore, the unknown parameters of the model could be estimated by conditional least squares or maximum likelihood methods.

## 3. Monitoring Procedure

In this section, we present a CUSUM chart for monitoring the ZIGINAR${}_{\mathrm{RC}}$(1) process. As this process is used to fit the number of crimes, an increase in the process mean usually means a deteriorating public security environment, and an increase in the process correlation usually means more re-offendings. Thus, our purpose is to detect the increasing of both mean shifts and correlation shifts in the ZIGINAR${}_{\mathrm{RC}}$(1) process. According to the model properties, the process mean is affected by the parameters θ and *p*, the correlation is affected by the parameters α and β. Let $\theta_{0},\;p_{0},\;\alpha_{0}$ and $\beta_{0}$ ($\theta_{1},\;p_{1},\;\alpha_{1}$ and $\beta_{1}$) denote the in-control (out-of-control) parameters of the processes, and $\mu_{0},\;\sigma_{0},\;\rho_{0}$ ($\mu_{1},\;\sigma_{1},\;\rho_{1}$) be the corresponding in-control (out-of-control) process mean, standard deviation and first-order correlation.

The CUSUM charts are commonly used charts in statistical process control, which were first proposed by Page [30]. The essential assumption underlying the design of CUSUM charts is that the process observations are independent (Montgomery [31], Alencar, Ho and Albarracin [32], Bourguignon, Medeiros, Fernandes and Ho [33]). While the violation of this major assumption seriously affects the monitoring performance of the charts (Harris and Ross [34], Triantafyllopoulos and Bersimis [35], Albarracin, Alencar and Ho [36]). Some authors have studied the performance of CUSUM charts for some integer-valued models (Weiß and Testik [23], Weiß and Testik [24], Yontay, Weiß, Testik and Bayindir [25], Rakitzis, Weiß and Castagliola [26], Li, Wang and Sun [27], Lee and Kim [37], Lee, Kim and Kim [38]).

**Scheme (The ZIGINAR${}_{RC}$(1) CUSUM chart).** *Let ${\left\{X_{t}\right\}}_{N_{0}}$ be a stationary ZIGINAR${}_{RC}$(1) process, the CUSUM statistics $C_{t}$ is defined as:*

$$C_{t}=\max\left(0,\;X_{t}-k+C_{t-1}\right),\;t=1,\;2,\cdots ,$$

*where k is a positive integer constant representing the reference ($k⩾\mu_{0}$). This chart is said to be out-of-control when $C_{t}$ falls outside the control limit h (h∈N), that is, $C_{t}>h$.*

The initial value of the CUSUM statistics is set equal to the integer constant $c_{0}$, i.e., $C_{0}=c_{0}$ with $c_{0}<h$. The performance evaluation of this chart is accomplished based on the average run length (ARL) measures, which is defined as the average number of points to be plotted on the chart until the first out-of-control signal triggers. As ${\left\{X_{t},C_{t}\right\}}_{t\in N_{0}}$ of the ZIGINAR${}_{\mathrm{RC}}$(1) process is a bivariate Markov chain, the Markov chain approach proposed by Brook and Evans [39] is adapted to evaluate the exact ARLs. Though this method has been described in detail in the relevant literature by Weiß [22], Weiß and Testik [23] and Weiß and Testik [24], we briefly introduce this method here for completeness. The reachable control region (CR) of ${\left\{X_{t},C_{t}\right\}}_{N_{0}}$ is given by

$$\begin{array}{cc} \mathrm{CR} & ≜\{\left(n,i\right)\in N_{0}\times \left\{0,\cdots ,h\right\}|\max\left(0,n-k+i\right)\in \left\{0,\cdots ,h\right\}\}\\ \; & =\{(n,i)|i\in \{0,\cdots ,h\},\;n\in \{\max(0,i+k-h),\cdots ,i+k\}\}. \end{array}$$

Obviously CR has a finite number of elements and could be ordered in a certain manner. The transition probability matrix of ${\left\{X_{t},C_{t}\right\}}_{N_{0}}$ is $Q^{⊤}≜{\left(p\left(n,j|m,i\right)\right)}_{(n,j),(m,i)\in \mathrm{CR}}$,

$$p\left(n,j|m,i\right)≜P\left(X_{t}=n,C_{t}=j|X_{t-1}=m,C_{t-1}=i\right)=I_{\left(j-\max(0,n-k+i)\right)}P\left(X_{t}=n|X_{t-1}=m\right).$$

The initial probabilities are

$$p\left(n,j|c_{0}\right)≜P\left(X_{1}=n,C_{1}=j|C_{0}=c_{0}\right)=I_{\left(j-\max\left(0,n-k+c_{0}\right)\right)}P\left(X_{1}=n\right).$$

The conditional probability that the run length of ${\left\{X_{t},C_{t}\right\}}_{N_{0}}$ equals *r* is defined by

$$p_{m,i}\left(r\right)≜P\left(\left(X_{r+1},C_{r+1}\right)\notin \mathrm{CR},\left(X_{r},C_{r}\right),\cdots ,\left(X_{2},C_{2}\right)\in \mathrm{CR}|\left(X_{1},C_{1}\right)=\left(m,i\right)\right),$$

where (m,i)∈CR. Let the vector $\mu_{\left(k\right)}$ denote the *k*-th factorial moments that ${\left(u_{\left(k\right)}\right)}_{m,i}≜\sum_{r=1}^{\infty }r_{\left(k\right)}p_{m,i}\left(r\right)$ where k⩾1 and $r_{\left(k\right)}≜r\times \left(r-1\right)\times \cdots \times \left(r-k+1\right)$. Then

$$p_{m,i}\left(r\right)=\sum_{(n,j)\in \mathrm{CR}}p_{n,j}\left(r-1\right)\times p\left(n,j|m,i\right),$$

$${\left(u_{\left(1\right)}\right)}_{m,i}=1+\sum_{(n,j)\in \mathrm{CR}}p\left(n,j|m,i\right)\times {\left(u_{\left(1\right)}\right)}_{n,j},\;\;i.e.,\;\;\left(I-Q\right)u_{\left(1\right)}=1.$$

The ARL is obtained as

$$\mathrm{ARL}=\sum_{(m,i)\in \mathrm{CR}}{\left(u_{\left(1\right)}\right)}_{m,i}\times p\left(m,i|c_{0}\right).$$

For simplicity we do not repeat the proof methods, see Weiß [22], Weiß and Testik [23] and Weiß and Testik [24] for more details. It is expected that an efficient chart possesses a large in-control ARL (denoted as ARL${}_{0}$) and a small out-of-control ARL. Along with the ARL, we also assess the performance of the charts through the standard deviation of run length (SDRL) suggested by Weiß [22]. The SDRL of the ZIGINAR${}_{\mathrm{RC}}$(1) CUSUM chart could again be computed efficiently by applying the Markov chain method. The second order factorial moments $u_{\left(2\right)}$ can be determined recursively from the relation $\left(I-Q\right)u_{\left(2\right)}=2{\mathrm{Qu}}_{\left(1\right)}$. Then the SDRL is

$$\mathrm{SDRL}=\sqrt{\sum_{(m,i)\in \mathrm{CR}}\left({\left(u_{\left(2\right)}\right)}_{m,i}+{\left(u_{\left(1\right)}\right)}_{m,i}\right)\times p\left(m,i|c_{0}\right)-{\mathrm{ARL}}^{2}}\;.$$

To implement the proposed monitoring scheme, the chart design pairs (h,k) need to be designed in advance. Generally, a fixed ARL${}_{0}$ value is set to be the target value, and (h,k) is set accordingly. Some guidelines for the choices of them will be given in the next section.

## 4. Computation Results

In this section, we evaluate the ZIGINAR${}_{\mathrm{RC}}$(1) CUSUM chart performance basing on extensive numerical experiments and presume that the parameters in this model have already been known. In practice, the in-control parameters need to be estimated from the data, as shown in the next section. We search for possible chart designs (integer (h,k) pairs) in order to adjust the ARL${}_{0}$ close to the target value. Here the target ARL${}_{0}$ value is set to be 370, which is commonly used in the statistical process monitoring domain. Meanwhile, the values of ARL and SDRL are calculated accurately by the Markov chain method, and we only show the results with two decimal places for simplicity. We first compute ARL${}_{0}$ and SDRL${}_{0}$ of the CUSUM chart for different in-control process parameters and initial values in Table 1. The process parameters are: $\theta_{0}=\left\{1,5\right\}$; $p_{0}=\left\{0.1,0.3\right\}$; $\alpha_{0}=\left\{0.5,0.8\right\}$; $\beta_{0}=\left\{0.5,0.8\right\}$. Furthermore, initial values are $c_{0}=\left\{0,3,6\right\}$. These chosen parameters could cover a broad range of different scenarios. Based on the results in Table 1, three important conclusions can be derived. First, it can be observed that when $c_{0}$ takes smaller value, the deviation of ARL${}_{0}$ and SDRL${}_{0}$ is small. When $c_{0}$ takes larger value, there might be a situation where the value of SDRL${}_{0}$ is significantly greater than the value of ARL${}_{0}$ (for example, ARL${}_{0}=409.42$, SDRL${}_{0}=442.13$ under $\left(\theta_{0},p_{0},\alpha_{0},\beta_{0},c_{0}\right)=\left(1,0.3,0.5,0.8,6\right)$). Thus, we assume that $c_{0}=0$ in the following studies to get better robust. Second, as the differences of the values between ARL and SDRL are small when $c_{0}=0$, we only use ARL as the measure in the following computations to save space. Last, the parameter $\theta_{0}$ shows a great influence on the selection of control designs (h,k), with a larger $\theta_{0}$ comes a larger pair of (h,k).

**Figure 2:**
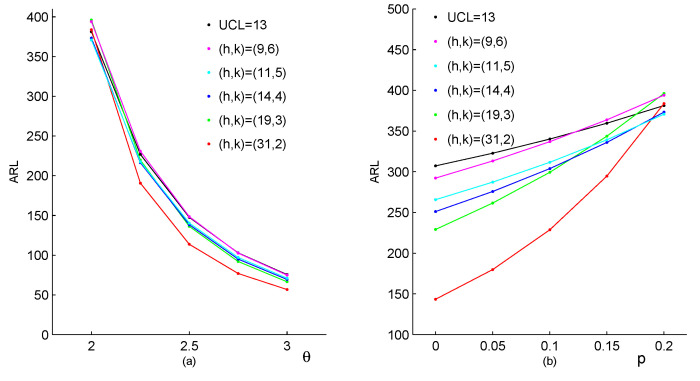
The performance of the Shewhart chart and the CUSUM chart to detect an increase in process mean, (**a**) changes only in θ, (**b**) changes only in *p*.

**Figure 3:**
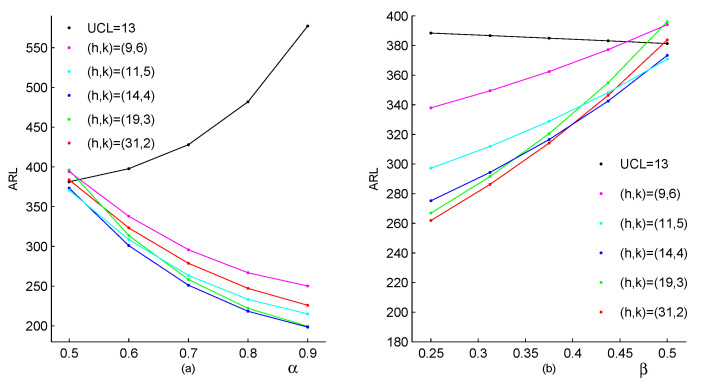
The performance of the Shewhart chart and the CUSUM chart to detect an increase in process first-order correlation, (**a**) changes only in α, (**b**) changes only in β.

Due to its simplicity, the conventional Shewhart chart is very popular in monitoring the process shifts. The upper limit for the Shewhart chart is denoted as UCL. For observations, when the value of the process ${\left\{X_{t}\right\}}_{N_{0}}$ exceeds the threshold value UCL ($X_{t}>UCL$), a fault is declared. Figure 2 and Figure 3 investigate the CUSUM method preliminarily by comparing it with the Shewhart method. In both of these figures, we assume that the in-control parameters are $\theta_{0}=2,\;p_{0}=0.2,\;\alpha_{0}=0.5$ and $\beta_{0}=0.5$, which are selected based on the real drug crime data in Section 5. According to these parameters, the CUSUM chart designs can be determined, respectively, as h=31,k=2 (corresponding ARL${}_{0}=383.74$); h=19,k=3 (ARL${}_{0}=396.12$); h=14,k=4 (ARL${}_{0}=373.27$); h=11,k=5 (ARL${}_{0}=370.77$); h=9,k=6 (ARL${}_{0}=394.03$). Furthermore, the Shewhart chart limit UCL=13 (ARL${}_{0}$=381.31) can be used. It should be noted that two types of changes are considered in Figure 2, which both lead to the upward mean shifts. The first type of changes occurs only in the parameter θ, with other parameters invariant, the results are listed in Figure 2a. Similarly, the second type of changes occurs only in the parameter *p*, with other parameters invariant, the results are in Figure 2b. From Figure 2, we can conclude that the CUSUM chart with the design h=31,k=2 outperforms the other CUSUM charts under most shifts, while the Shewhart chart performs worst among them. For the upward correlation shift scenarios, ARL values under two types of the parameter changes are displayed in Figure 3. The first one considers changes only in the parameter α, and the second one considers changes only in the parameter β. For each scenario, the Shewhart chart performs increase ratio of ARL with the increase of first-order correlation $\rho_{X}$, and the CUSUM chart has the better behaviour in the figure. In a comprehensive view, the conventional Shewhart chart is insensitive for upward mean shifts caused by changes in parameter *p*, and fails to detect shifts in the correlation. While the proposed CUSUM chart could overcome these limitations and has superiorities in various coefficient shifts compared with the Shewhart chart. From the figures, we can also conclude that the smaller the value of *k*, the more sensitive the CUSUM chart is. As the constraint $k⩾\mu_{0}$ is required to make the chart reasonable, it is natural to recommend $k=\lceil \mu_{0}\rceil$ (the smallest integer no less than $\mu_{0}$), then we aim to select the value of *h* such that ARL${}_{0}$ is close to 370. Now the computations of the CUSUM chart are extended to general cases with designs of experiments as follow.

In Table 2, Table 3 and Table 4, we focus on situations that there are increasing shifts in process mean, and the correlation remains the same. Each in-control parameter has three levels: $\theta_{0}=\left\{1,3,5\right\}$; $p_{0}=\left\{0.1,0.2,0.3\right\}$; $\alpha_{0}=\left\{0.5,0.6,0.7\right\}$; $\beta_{0}=\left\{0.5,0.6,0.7\right\}$. We consider the case when the changes only occur in θ, this is the most common case. The out-of-control process mean is $\mu_{1}=\mu_{0}+\delta \sigma_{0}$, the shift size δ considers potential values in set {0.5,1,1.5,6}. The usual relative deviation (in %) in ARL is defined as dev${}^{\left(\%\right)}$ = 100%× (ARL-ARL${}_{0}$)/ARL${}_{0}$ (Weiß and Testik [24]). From Table 2, Table 3, Table 4, we can conclude that the ZIGINAR${}_{\mathrm{RC}}$(1) CUSUM chart performs quite well in detecting upward mean shifts for all scenarios. For the small shift size of δ=0.5, the CUSUM chart is efficient with the minimum 86.38% drop of ARL and the maximum 91.23% drop of ARL. Take larger δ for another illustration (δ=1), the drop of ARL is at least 92.81%, and up to 96.03% at most. It can also be obtained that δ has to be at least 6 to get an immediate signal with the out-of-control ARL closer to 1. In addition, extensive computation results show that the in-control parameters ($\theta_{0},p_{0},\alpha_{0},\beta_{0}$) have little effect on the better performance of the CUSUM chart to detect the mean shifts.

The computation study in Table 5 and Table 6 concerns the upward shifts in the process correlation. Two levels are accepted for each in-control parameter: $\theta_{0}=\left\{1,5\right\}$; $p_{0}=\left\{0.1,0.2\right\}$; $\alpha_{0}=\left\{0.5,0.6\right\}$; $\beta_{0}=\left\{0.7,0.8\right\}$. Two types of out-of-control pattern are considered here for comprehensive investigation, the first type is that the upward changes only exist in α (shown in Table 5), and the second type is that the downward changes only exist in β (shown Table 6). In Table 5, the shifts in the magnitude $\delta_{\alpha }=\alpha_{1}-\alpha_{0}$ are from the set {0.1,0.2,0.3}. The results imply that the performance of CUSUM chart fluctuates greatly in detecting correlation shifts caused only by α. To be specific, when $\delta_{\alpha }$ is 0.3, dev${}^{\left(\%\right)}$ ranges from −6.81% to −23.68%. Another finding based on the design of experiments in Table 5 is that both a smaller $\theta_{0}$ and a smaller $\beta_{0}$ could slightly improve detection efficiency, while $p_{0}$ and $\alpha_{0}$ could not. In Table 6, the shifts magnitude $\delta_{\beta }=\beta_{1}-\beta_{0}$ are from the set {−0.1,−0.2,−0.3}. From Table 6, we can see the CUSUM chart is more efficient in detecting the correlation shifts caused by β. As the absolute value of $\delta_{\beta }$ gets bigger, the decreasing proportion of ARL gradually increased. When $\delta_{\beta }=-0.3$, dev${}^{\left(\%\right)}$ ranges from −21.3% to −40.39%. Meanwhile, we can further conclude that a smaller $\theta_{0}$ and a larger $\alpha_{0}$ often lead to better chart performance, and $p_{0},\beta_{0}$ have little influence. Based on all the analysis above in this paragraph, we can further conclude that a smaller $\theta_{0}$ and a larger value of initial correlation $\rho_{0}$ are helpful to detect the correlation shifts. Furthermore, that we cannot get an immediate signal when only correlation shifts occur.

## 5. Analyses of Drug Crime Count Time Series

**Figure 4:**
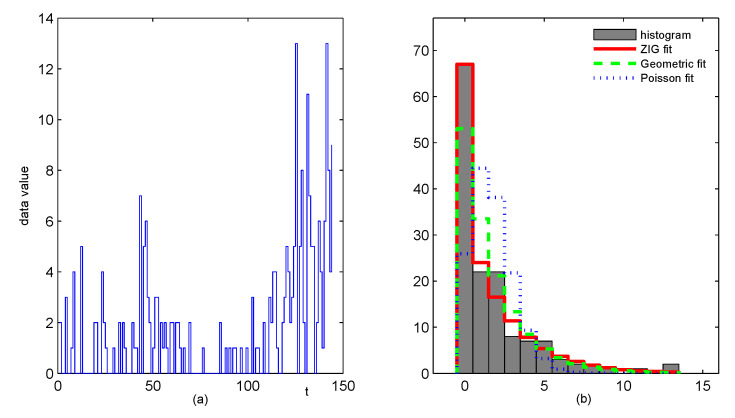
The plots about the zero-inflated drug crime series, (**a**) the sample path, (**b**) the histogram with ZIG fit, Geometric fit and Poisson fit.

**Figure 5:**
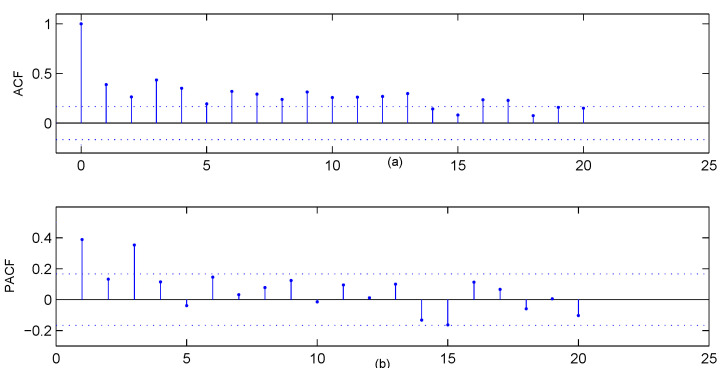
The plots about the zero-inflated drug crime series, (**a**) the ACF plot, (**b**) the PACF plot.

In this section, we present a case study of crime count data in Pittsburgh. The data set contains multiple crime types, such as arson, drink-driving, robbery and so on. Monitoring of crime data is needed not only for early warnings of the organised crime, but also for assessments of the social security environment. For the crime data, the readers can download it from the Forecasting Principles site (http://www.forecastingprinciples.com, accessed on 20 March 2021), or email to the corresponding author to access. The subset we analyse is a monthly drug use count data collected from the 56th police car beat, which contains 144 observations from January 1990 to December 2001. There are 67 zeros in this drug use data (the proportion up to 46.53%), which have the greatest proportion among the other values for the data series. The sample mean, variance and first-order autocorrelation of the data are 1.7153, 6.4289 and 0.3886, respectively, which show strong overdispersion and autocorrelation. The sample path and the histogram of the series are in Figure 4. The histograms of estimated ZIG distribution, estimated Geometric distribution and estimated Poisson distribution are also given in Figure 4b, which indicate that the ZIG marginal is the most appropriate to describe the data. The sample autocorrelation function (ACF) and the sample partial autocorrelation function (PACF) in Figure 5 reveal that the series most likely comes from an AR-type process of order 3. While our intention is to illustrate the implementation of the proposed control chart, we will employ the first-order INAR models that are widely studied and applied in the literature. The consideration of more complex models will be left for future study.

**Figure 6:**
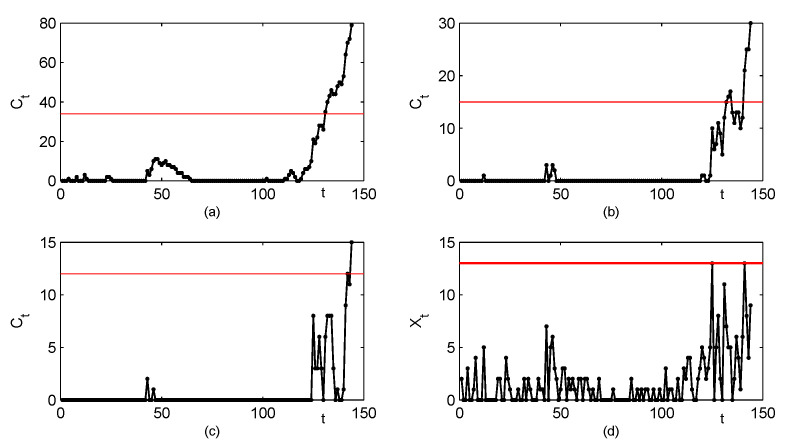
The control charts for the zero-inflated drug crime series, the CUSUM charts with designs (**a**) h=34,k=2, (**b**) h=15,k=4, (**c**) h=12,k=5, and (**d**) the Shewhart chart with UCL=13.

Except for the ZIGINAR${}_{\mathrm{RC}}$(1) model, some competitive models are also applied to the time series, such as Poisson INAR(1) (Al-Osh and Alzaid [1]), GINAR(1) (Alzaid and Al-Osh [7]), ZINAR(1) (Jazi, Jones and Lai [9]), ZMGINAR(1) (Barreto-Souza [11]), NGINAR(1) (Ristić, Bakouch and Nastić [12]), ZIMINAR(1) (Li, Wang and Zhang [14]). The Akaike Information Criterion (AIC) and Bayesian Information Criterion (BIC) are suggested to evaluate these models. Numerical results in Table 7 show that the ZIGINAR${}_{\mathrm{RC}}$(1) process has the best overall performance, compared with its competitors. Therefore, we assume that the drug crime data is from the ZIGINAR${}_{\mathrm{RC}}$(1) model and the estimated parameters in Table 7 are used in the process control procedures. Based on the computation results in Section 4, the CUSUM chart with designs h=34,k=2 (corresponding ARL${}_{0}=364.44$) is the best choice, which is shown in Figure 6a. For comparison, we also present CUSUM control charts with designs h=15,k=4 (ARL${}_{0}=358.40$) in Figure 6b, designs h=12,k=5 (ARL${}_{0}=372.28$) in Figure 6c, and the Shewhart chart with control limit UCL=13 (ARL${}_{0}=340.25$) in Figure 6d. We observe that all the CUSUM charts give out-of-control signals, while there is no outliers in the Shewhart chart. Because the Shewhart chart has been proved to be less effective than the CUSUM chart, the drug crime data set seems to be out-of-control with increasing mean shifts or increasing correlation shifts, and some investigation should be done for further explanation. The CUSUM control charts with three designs also display different detection efficiencies. The CUSUM chart with k=2 first signals at t=131 following with continuous alarms as *t* increases. The signals of the CUSUM chart with k=4 are first given at t=133, then go back below the control limit over a period of time, and come again at t=141. While the outlier of the CUSUM chart with k=5 occurs at t=144. The analysis above proves again that the CUSUM chart design $k=\lceil \mu_{0}\rceil$ is the most effective in practice.

## 6. Conclusions

In this paper, we have made contributions on monitoring the zero-inflated autocorrelated count data which can be described by an INAR(1) process with random coefficient. ARL and SDRL are adopted to be the measures and calculated by the Markov chain approach. The design parameter *k* in the CUSUM chart is proved to have great influence on monitoring efficiency, and the smallest integer no less than $\mu_{0}$ is the recommended value of *k*. The proposed ZIGINAR${}_{\mathrm{RC}}$(1)CUSUM chart is proved to have superiorities in various coefficient shifts compared with the conventional Shewhart chart. Computation results also show that the CUSUM chart performs quite well in detecting upward mean shifts, and shows fluctuation in detecting upward correlation shifts. Based on the design of experiment, we also find that a larger value of initial correlation $\rho_{0}$ is helpful to detect the correlation shifts. An immediate signal occurs after large upward mean shifts, while does not occur when only correlation shifts exist.

There are some possible topics for our future research. First, we can consider a different monitoring scheme for the ZIGINAR${}_{\mathrm{RC}}$(1) process and conduct a comparison study. Second, we can explore the monitoring of *p*-th random coefficient INAR model, which is suitable for the count data with high order dependence. Third, we can study a multivariate INAR(1) process with random coefficient to continuously monitor the serial correlated counts that we are interested in. 

## Figures and Tables

**Table 1:** ARL${}_{0}$ and SDRL${}_{0}$ of the CUSUM chart for various $\theta_{0},p_{0},\alpha_{0},\beta_{0}$ and $c_{0}$.

$\left(\theta_{0},\;p_{0},\;\alpha_{0},\;\beta_{0}\right)$	(h,k)	(c${}_{0}$, ARL${}_{0}$, SDRL${}_{0}$)	(c${}_{0}$, ARL${}_{0}$, SDRL${}_{0}$)	(c${}_{0}$, ARL${}_{0}$, SDRL${}_{0}$)
(1, 0.1, 0.5, 0.5)	(9, 2)	(0, 340.55, 339)	(3, 336.84, 338.98)	(6, 322.88, 338.52)
(1, 0.1, 0.5, 0.8)	(8, 2)	(0, 428.55, 427.38)	(3, 423.5, 427.34)	(6, 398.83, 426.33)
(1, 0.1, 0.8, 0.5)	(12, 2)	(0, 368.36, 366.45)	(3, 365.76, 366.43)	(6, 358.76, 366.3)
(1, 0.1, 0.8, 0.8)	(9, 2)	(0, 428.69, 427.42)	(3, 424.79, 427.4)	(6, 408.35, 426.91)
(1, 0.3, 0.5, 0.5)	(8, 2)	(0, 385.69, 384.65)	(3, 381.9, 384.62)	(6, 365.66, 384.11)
(1, 0.3, 0.5, 0.8)	(7, 2)	(0, 444.16, 443.51)	(3, 438.89, 443.47)	(6, 409.42, 442.13)
(1, 0.3, 0.8, 0.5)	(10, 2)	(0, 359.91, 358.61)	(3, 357.26, 358.6)	(6, 349.32, 358.44)
(1, 0.3, 0.8, 0.8)	(8, 2)	(0, 469.37, 468.53)	(3, 465.3, 468.51)	(6, 446.23, 467.94)
(5, 0.1, 0.5, 0.5)	(60, 6)	(0, 379.61, 371.51)	(3, 379.07, 371.51)	(6, 378.25, 371.51)
(5, 0.1, 0.5, 0.8)	(49, 6)	(0, 376.02, 369.17)	(3, 375.4, 369.17)	(6, 374.38, 369.16)
(5, 0.1, 0.8, 0.5)	(75, 6)	(0, 371.37, 363.76)	(3, 370.91, 363.76)	(6, 370.28, 363.75)
(5, 0.1, 0.8, 0.8)	(54, 6)	(0, 378.57, 372.12)	(3, 378.01, 372.12)	(6, 377.14, 372.11)
(5, 0.3, 0.5, 0.5)	(46, 6)	(0, 383.15, 379.48)	(3, 382.64, 379.47)	(6, 381.86, 379.47)
(5, 0.3, 0.5, 0.8)	(38, 6)	(0, 386.29, 383.42)	(3, 385.68, 383.42)	(6, 384.7, 383.42)
(5, 0.3, 0.8, 0.5)	(59, 6)	(0, 378.46, 374.67)	(3, 378.04, 374.67)	(6, 377.45, 374.67)
(5, 0.3, 0.8, 0.8)	(42, 6)	(0, 379.79, 377.04)	(3, 379.26, 377.04)	(6, 378.45, 377.03)

**Table 2:** ARL profiles of the CUSUM chart versus mean shifts under $\theta_{0}=1$.

Process Parameters						ARL (dev${}^{\left(\%\right)}$)				
$p_{0}$	$\alpha_{0}$	$\beta_{0}$	$\mu_{0}$	h	k	δ=0	δ=0.5	δ=1	δ=1.5	δ=6
0.1	0.5	0.5	0.9	22	1	348.22	38.62	19.31	12.94	3.44
							(−88.91%)	(−94.45%)	(−96.28%)	(−99.01%)
0.1	0.5	0.6	0.9	21	1	357.74	36.77	18.28	12.21	3.21
							(−89.72%)	(−94.89%)	(−96.59%)	(−99.1%)
0.1	0.5	0.7	0.9	20	1	365.71	34.91	17.27	11.51	3
							(−90.45%)	(−95.28%)	(−96.85%)	(−99.18%)
0.1	0.6	0.5	0.9	24	1	370.79	42.46	21.23	14.23	3.78
							(−88.55%)	(−94.27%)	(−96.16%)	(−98.98%)
0.1	0.6	0.6	0.9	22	1	358.58	38.82	19.32	12.91	3.39
							(−89.17%)	(−94.61%)	(−96.4%)	(−99.05%)
0.1	0.6	0.7	0.9	21	1	378.05	36.86	18.23	12.15	3.16
							(−90.25%)	(−95.18%)	(−96.79%)	(−99.16%)
0.1	0.7	0.5	0.9	25	1	358.79	44.75	22.43	15.04	3.99
							(−87.53%)	(−93.75%)	(−95.81%)	(−98.89%)
0.1	0.7	0.6	0.9	23	1	359.01	40.95	20.39	13.63	3.57
							(−88.59%)	(−94.32%)	(−96.2%)	(−99.01%)
0.1	0.7	0.7	0.9	21	1	351.73	37.15	18.41	12.28	3.19
							(−89.44%)	(−94.77%)	(−96.51%)	(−99.09%)
0.2	0.5	0.5	0.8	18	1	371.21	37.8	18.14	12.01	3.26
							(−89.82%)	(−95.11%)	(−96.76%)	(−99.12%)
0.2	0.5	0.6	0.8	17	1	378.49	35.72	17	11.22	3.01
							(−90.56%)	(−95.51%)	(−97.04%)	(−99.2%)
0.2	0.5	0.7	0.8	16	1	382.96	33.57	15.88	10.45	2.77
							(−91.23%)	(−95.85%)	(−97.27%)	(−99.28%)
0.2	0.6	0.5	0.8	19	1	360.14	40.29	19.42	12.87	3.49
							(−88.81%)	(−94.61%)	(−96.43%)	(−99.03%)
0.2	0.6	0.6	0.8	18	1	382.06	38.15	18.17	11.99	3.2
							(−90.01%)	(−95.24%)	(−96.86%)	(−99.16%)
0.2	0.6	0.7	0.8	16	1	346.1	33.8	16.06	10.58	2.81
							(−90.23%)	(−95.36%)	(−96.94%)	(−99.19%)
0.2	0.7	0.5	0.8	20	1	349.23	42.95	20.77	13.77	3.71
							(−87.7%)	(−94.05%)	(−96.06%)	(−98.94%)
0.2	0.7	0.6	0.8	18	1	338.16	38.56	18.49	12.21	3.25
							(−88.6%)	(−94.53%)	(−96.39%)	(−99.04%)
0.2	0.7	0.7	0.8	17	1	365.85	36.24	17.17	11.29	2.98
							(−90.09%)	(−95.31%)	(−96.91%)	(−99.19%)
0.3	0.5	0.5	0.7	15	1	376.37	37.77	17.57	11.54	3.25
							(−89.96%)	(−95.33%)	(−96.93%)	(−99.14%)
0.3	0.5	0.6	0.7	14	1	374.56	35.37	16.31	10.67	2.97
							(−90.56%)	(−95.65%)	(−97.15%)	(−99.21%)
0.3	0.5	0.7	0.7	13	1	368.03	32.86	15.04	9.81	2.71
							(−91.07%)	(−95.91%)	(−97.33%)	(−99.26%)
0.3	0.6	0.5	0.7	16	1	369.03	40.69	19.04	12.51	3.5
							(−88.97%)	(−94.84%)	(−96.61%)	(−99.05%)
0.3	0.6	0.6	0.7	14	1	323.29	35.58	16.61	10.89	3.03
							(−88.99%)	(−94.86%)	(−96.63%)	(−99.06%)
0.3	0.6	0.7	0.7	13	1	328.19	33.03	15.25	9.96	2.75
							(−89.94%)	(−95.35%)	(−96.97%)	(−99.16%)
0.3	0.7	0.5	0.7	17	1	360.19	43.81	20.59	13.53	3.75
							(−87.84%)	(−94.28%)	(−96.24%)	(−98.96%)
0.3	0.7	0.6	0.7	15	1	335.26	38.66	18	11.79	3.24
							(−88.47%)	(−94.63%)	(−96.48%)	(−99.03%)
0.3	0.7	0.7	0.7	14	1	357.18	36.06	16.52	10.76	2.93
							(−89.9%)	(−95.37%)	(−96.99%)	(−99.18%)

**Table 3:** ARL profiles of the CUSUM chart versus mean shifts under $\theta_{0}=3$.

Process Parameters						ARL (dev${}^{\left(\%\right)}$)				
$p_{0}$	$\alpha_{0}$	$\beta_{0}$	$\mu_{0}$	h	k	δ=0	δ=0.5	δ=1	δ=1.5	δ=6
0.1	0.5	0.5	2.7	54	3	364.48	38.52	19.06	12.72	3.37
							(−89.43%)	(−94.77%)	(−96.51%)	(−99.08%)
0.1	0.5	0.6	2.7	51	3	366.54	36.32	17.87	11.9	3.11
							(−90.09%)	(−95.12%)	(−96.75%)	(−99.15%)
0.1	0.5	0.7	2.7	48	3	365.43	34.07	16.69	11.09	2.87
							(−90.68%)	(−95.43%)	(−96.97%)	(−99.21%)
0.1	0.6	0.5	2.7	58	3	373.32	41.8	20.71	13.83	3.66
							(−88.8%)	(−94.45%)	(−96.3%)	(−99.02%)
0.1	0.6	0.6	2.7	54	3	374.45	38.8	19.09	12.71	3.32
							(−89.64%)	(−94.9%)	(−96.61%)	(−99.11%)
0.1	0.6	0.7	2.7	50	3	369.98	35.75	17.51	11.63	3.01
							(−90.34%)	(−95.27%)	(−96.86%)	(−99.19%)
0.1	0.7	0.5	2.7	61	3	365.9	44.55	22.1	14.76	3.89
							(−87.82%)	(−93.96%)	(−95.97%)	(−98.94%)
0.1	0.7	0.6	2.7	56	3	366.07	40.68	20.03	13.33	3.47
							(−88.89%)	(−94.53%)	(−96.36%)	(−99.05%)
0.1	0.7	0.7	2.7	52	3	374.26	37.49	18.34	12.17	3.14
							(−89.98%)	(−95.1%)	(−96.75%)	(−99.16%)
0.2	0.5	0.5	2.4	43	3	371.94	36.87	17.43	11.48	3.1
							(−90.09%)	(−95.31%)	(−96.91%)	(−99.17%)
0.2	0.5	0.6	2.4	40	3	363.7	34.31	16.11	10.58	2.82
							(−90.57%)	(−95.57%)	(−97.09%)	(−99.22%)
0.2	0.5	0.7	2.4	38	3	377.02	32.59	15.16	9.92	2.62
							(−91.36%)	(−95.98%)	(−97.37%)	(−99.31%)
0.2	0.6	0.5	2.4	46	3	368.96	39.91	18.93	12.47	3.35
							(−89.18%)	(−94.87%)	(−96.62%)	(−99.09%)
0.2	0.6	0.6	2.4	43	3	379.62	37.34	17.5	11.48	3.04
							(−90.16%)	(−95.39%)	(−96.98%)	(−99.2%)
0.2	0.6	0.7	2.4	39	3	360.73	33.72	15.72	10.29	2.7
							(−90.65%)	(−95.64%)	(−97.15%)	(−99.25%)
0.2	0.7	0.5	2.4	49	3	363.38	43.17	20.52	13.51	3.6
							(−88.12%)	(−94.35%)	(−96.28%)	(−99.01%)
0.2	0.7	0.6	2.4	45	3	370.51	39.59	18.57	12.17	3.2
							(−89.31%)	(−94.99%)	(−96.72%)	(−99.14%)
0.2	0.7	0.7	2.4	41	3	369.61	35.87	16.68	10.89	2.84
							(−90.3%)	(−95.49%)	(−97.05%)	(−99.23%)
0.3	0.5	0.5	2.1	36	3	380.79	36.83	16.87	11.01	3.08
							(−90.33%)	(−95.57%)	(−97.11%)	(−99.19%)
0.3	0.5	0.6	2.1	33	3	357.51	33.81	15.39	10.01	2.78
							(−90.54%)	(−95.7%)	(−97.2%)	(−99.22%)
0.3	0.5	0.7	2.1	31	3	360.6	31.82	14.33	9.28	2.55
							(−91.18%)	(−96.03%)	(−97.43%)	(−99.29%)
0.3	0.6	0.5	2.1	38	3	358.5	39.26	18.15	11.86	3.3
							(−89.05%)	(−94.94%)	(−96.69%)	(−99.08%)
0.3	0.6	0.6	2.1	35	3	355.89	36.32	16.56	10.76	2.96
							(−89.79%)	(−95.35%)	(−96.98%)	(−99.17%)
0.3	0.6	0.7	2.1	33	3	378.11	34.37	15.41	9.96	2.71
							(−90.91%)	(−95.92%)	(−97.37%)	(−99.28%)
0.3	0.7	0.5	2.1	41	3	359.33	43.13	19.97	13.03	3.58
							(−88%)	(−94.44%)	(−96.37%)	(−99%)
0.3	0.7	0.6	2.1	38	3	377.93	40.22	18.23	11.81	3.2
							(−89.36%)	(−95.18%)	(−96.88%)	(−99.15%)
0.3	0.7	0.7	2.1	34	3	362.28	35.83	16.08	10.38	2.8
							(−90.11%)	(−95.56%)	(−97.13%)	(−99.23%)

**Table 4:** ARL profiles of the CUSUM chart versus mean shifts under $\theta_{0}=5$.

Process Parameters						ARL (dev${}^{\left(\%\right)}$)				
$p_{0}$	$\alpha_{0}$	$\beta_{0}$	$\mu_{0}$	h	k	δ=0	δ=0.5	δ=1	δ=1.5	δ=6
0.1	0.5	0.5	4.5	85	5	365.12	38.31	18.91	12.61	3.33
							(−89.51%)	(−94.82%)	(−96.55%)	(−99.09%)
0.1	0.5	0.6	4.5	80	5	364.27	36	17.67	11.76	3.07
							(−90.12%)	(−95.15%)	(−96.77%)	(−99.16%)
0.1	0.5	0.7	4.5	76	5	371.67	34.09	16.64	11.04	2.86
							(−90.83%)	(−95.52%)	(−97.03%)	(−99.23%)
0.1	0.6	0.5	4.5	91	5	370.55	41.46	20.5	13.68	3.61
							(−88.81%)	(−94.47%)	(−96.31%)	(−99.03%)
0.1	0.6	0.6	4.5	85	5	374.72	38.61	18.95	12.6	3.29
							(−89.7%)	(−94.94%)	(−96.64%)	(−99.12%)
0.1	0.6	0.7	4.5	79	5	373.87	35.71	17.43	11.56	2.99
							(−90.45%)	(−95.34%)	(−96.91%)	(−99.2%)
0.1	0.7	0.5	4.5	97	5	373.34	44.81	22.15	14.77	3.89
							(−88%)	(−94.07%)	(−96.04%)	(−98.96%)
0.1	0.7	0.6	4.5	89	5	373.74	40.89	20.06	13.34	3.47
							(−89.06%)	(−94.63%)	(−96.43%)	(−99.07%)
0.1	0.7	0.7	4.5	81	5	365.37	36.94	18.03	11.96	3.08
							(−89.89%)	(−95.07%)	(−96.73%)	(−99.16%)
0.2	0.5	0.5	4	118	4	373.44	45.69	24.01	16.42	4.62
							(−87.77%)	(−93.57%)	(−95.6%)	(−98.76%)
0.2	0.5	0.6	4	112	4	369.47	43.25	22.65	15.45	4.3
							(−88.29%)	(−93.87%)	(−95.82%)	(−98.84%)
0.2	0.5	0.7	4	107	4	369.02	41.18	21.48	14.63	4.03
							(−88.84%)	(−94.18%)	(−96.04%)	(−98.91%)
0.2	0.6	0.5	4	122	4	366.95	47.66	25.12	17.21	4.86
							(−87.01%)	(−93.15%)	(−95.31%)	(−98.68%)
0.2	0.6	0.6	4	115	4	365.11	44.74	23.47	16.03	4.47
							(−87.75%)	(−93.57%)	(−95.61%)	(−98.78%)
0.2	0.6	0.7	4	110	4	371.48	42.56	22.22	15.14	4.17
							(−88.54%)	(−94.02%)	(−95.92%)	(−98.88%)
0.2	0.7	0.5	4	128	4	371.06	50.55	26.69	18.29	5.17
							(−86.38%)	(−92.81%)	(−95.07%)	(−98.61%)
0.2	0.7	0.6	4	120	4	372.35	47.07	24.71	16.88	4.71
							(−87.36%)	(−93.36%)	(−95.47%)	(−98.74%)
0.2	0.7	0.7	4	112	4	368.48	43.62	22.79	15.53	4.28
							(−88.16%)	(−93.82%)	(−95.79%)	(−98.84%)
0.3	0.5	0.5	3.5	80	4	367.77	38.09	19.11	12.93	3.77
							(−89.64%)	(−94.8%)	(−96.48%)	(−98.97%)
0.3	0.5	0.6	3.5	75	4	363.52	35.68	17.79	12	3.45
							(−90.18%)	(−95.11%)	(−96.7%)	(−99.05%)
0.3	0.5	0.7	3.5	71	4	367.7	33.69	16.7	11.23	3.2
							(−90.84%)	(−95.46%)	(−96.95%)	(−99.13%)
0.3	0.6	0.5	3.5	85	4	366.71	40.93	20.6	13.95	4.06
							(−88.84%)	(−94.38%)	(−96.2%)	(−98.89%)
0.3	0.6	0.6	3.5	79	4	366.41	37.96	18.95	12.78	3.67
							(−89.64%)	(−94.83%)	(−96.51%)	(−99%)
0.3	0.6	0.7	3.5	74	4	372.31	35.4	17.55	11.8	3.35
							(−90.49%)	(−95.29%)	(−96.83%)	(−99.1%)
0.3	0.7	0.5	3.5	90	4	364.09	43.96	22.17	15.02	4.35
							(−87.93%)	(−93.91%)	(−95.87%)	(−98.81%)
0.3	0.7	0.6	3.5	83	4	368.41	40.36	20.16	13.59	3.88
							(−89.04%)	(−94.53%)	(−96.31%)	(−98.95%)
0.3	0.7	0.7	3.5	76	4	365.16	36.7	18.2	12.23	3.46
							(−89.95%)	(−95.02%)	(−96.65%)	(−99.05%)

**Table 5:** ARL profiles of the CUSUM chart versus correlation shifts caused by α.

Process Parameters							ARL (dev${}^{\left(\%\right)}$)			
$\theta_{0}$	$p_{0}$	$\alpha_{0}$	$\beta_{0}$	$\rho_{0}$	h	k	$\delta_{\alpha }=0$	$\delta_{\alpha }=0.1$	$\delta_{\alpha }=0.2$	$\delta_{\alpha }=0.3$
1	0.1	0.5	0.7	0.15	20	1	365.71	339.16	316.72	298.07
								(−7.26%)	(−13.4%)	(−18.5%)
1	0.1	0.5	0.8	0.1	19	1	371.95	353.06	336.45	322.04
								(−5.08%)	(−9.54%)	(−13.42%)
1	0.1	0.6	0.7	0.18	21	1	378.05	351.73	329.83	311.98
								(−6.96%)	(−12.75%)	(−17.48%)
1	0.1	0.6	0.8	0.12	19	1	353.06	336.45	322.04	309.72
								(−4.7%)	(−8.79%)	(−12.28%)
1	0.2	0.5	0.7	0.15	16	1	382.96	346.1	316.19	292.28
								(−9.63%)	(−17.44%)	(−23.68%)
1	0.2	0.5	0.8	0.1	15	1	384.25	357.92	335.41	316.44
								(−6.85%)	(−12.71%)	(−17.65%)
1	0.2	0.6	0.7	0.18	16	1	346.1	316.19	292.28	273.5
								(−8.64%)	(−15.55%)	(−20.98%)
1	0.2	0.6	0.8	0.12	15	1	357.92	335.41	316.44	300.62
								(−6.29%)	(−11.59%)	(−16.01%)
5	0.1	0.5	0.7	0.15	76	5	371.67	340.88	315.5	295.25
								(−8.28%)	(−15.11%)	(−20.56%)
5	0.1	0.5	0.8	0.1	72	5	377.08	355.15	336.23	320.32
								(−5.82%)	(−10.83%)	(−15.05%)
5	0.1	0.6	0.7	0.18	79	5	373.87	344.75	321.44	303.45
								(−7.79%)	(−14.02%)	(−18.84%)
5	0.1	0.6	0.8	0.12	73	5	367.42	347.51	330.75	316.97
								(−5.42%)	(−9.98%)	(−13.73%)
5	0.2	0.5	0.7	0.15	107	4	369.02	353.52	339.82	328.1
								(−4.2%)	(−7.91%)	(−11.09%)
5	0.2	0.5	0.8	0.1	103	4	372.99	362.65	353.34	345.14
								(−2.77%)	(−5.27%)	(−7.47%)
5	0.2	0.6	0.7	0.18	110	4	371.48	356.88	344.32	333.97
								(−3.93%)	(−7.31%)	(−10.1%)
5	0.2	0.6	0.8	0.12	104	4	369.05	359.52	351.13	343.92
								(−2.58%)	(−4.86%)	(−6.81%)

**Table 6:** ARL profiles of the CUSUM chart versus correlation shifts caused by β.

Process Parameters							ARL (dev${}^{\left(\%\right)}$)			
$\theta_{0}$	$p_{0}$	$\alpha_{0}$	$\beta_{0}$	$\rho_{0}$	h	k	$\delta_{\alpha }=0$	$\delta_{\alpha }=0.1$	$\delta_{\alpha }=0.2$	$\delta_{\alpha }=0.3$
1	0.1	0.5	0.7	0.15	20	1	365.71	321.34	284.33	252.99
								(−12.13%)	(−22.25%)	(−30.82%)
1	0.1	0.5	0.8	0.1	19	1	371.95	325.82	287.74	255.77
								(−12.4%)	(−22.64%)	(−31.24%)
1	0.1	0.6	0.7	0.18	21	1	378.05	324.46	281.01	245.09
								(−14.18%)	(−25.67%)	(−35.17%)
1	0.1	0.6	0.8	0.12	19	1	353.06	303.31	263.34	230.49
								(−14.09%)	(−25.41%)	(−34.72%)
1	0.2	0.5	0.7	0.15	16	1	382.96	325.3	280.11	243.87
								(−15.06%)	(−26.86%)	(−36.32%)
1	0.2	0.5	0.8	0.1	15	1	384.25	324.54	278.48	241.96
								(−15.54%)	(−27.53%)	(−37.03%)
1	0.2	0.6	0.7	0.18	16	1	346.1	288.47	244.5	209.96
								(−16.65%)	(−29.36%)	(−39.34%)
1	0.2	0.6	0.8	0.12	15	1	357.92	295.66	249.23	213.36
								(−17.39%)	(−30.37%)	(−40.39%)
5	0.1	0.5	0.7	0.15	76	5	371.67	322.61	282.59	249.42
								(−13.2%)	(−23.97%)	(−32.89%)
5	0.1	0.5	0.8	0.1	72	5	377.08	325.96	284.72	250.82
								(−13.56%)	(−24.49%)	(−33.48%)
5	0.1	0.6	0.7	0.18	79	5	373.87	316.52	271.1	234.34
								(−15.34%)	(−27.49%)	(−37.32%)
5	0.1	0.6	0.8	0.12	73	5	367.42	310.19	265.46	229.57
								(−15.58%)	(−27.75%)	(−37.52%)
5	0.2	0.5	0.7	0.15	107	4	369.02	340.45	313.89	289.18
								(−7.74%)	(−14.94%)	(−21.64%)
5	0.2	0.5	0.8	0.1	103	4	372.99	344.5	318.09	293.53
								(−7.64%)	(−14.72%)	(−21.3%)
5	0.2	0.6	0.7	0.18	110	4	371.48	337.45	306.12	277.2
								(−9.16%)	(−17.59%)	(−25.38%)
5	0.2	0.6	0.8	0.12	104	4	369.05	336.01	305.7	277.78
								(−8.95%)	(−17.17%)	(−24.73%)

**Table 7:** The estimated parameters, AIC and BIC of candidate models.

Model	Estimated Parameters		AIC	BIC
Poisson INAR(1)	$\hat{\lambda }=1.2806$	$\hat{\alpha }=0.2614$	625.787	631.7266
GINAR(1)	$\hat{p}=0.3581$	$\hat{\alpha }=0.2005$	507.0551	512.9947
NGINAR(1)	$\hat{\mu }=1.7925$	$\hat{\alpha }=0.3028$	502.6322	508.5718
ZINAR(1)	$\hat{\alpha }=0.2157$	$\hat{\lambda }=2.9131$	527.1981	536.1075
	$\hat{p}=0.5351$			
ZMGINAR(1)	$\hat{\mu }=1.9712$	$\hat{\alpha }=0.29$	501.7477	510.6571
	$\hat{\pi }=0.1197$			
ZIMINAR(1)	$\hat{\alpha }=0.001$	$\hat{\beta }=0.6731$	503.457	518.3061
	$\hat{p}=0.4101$	$\hat{\lambda }=2.2564$		
	$\hat{\rho }=0.548$			
ZIGINAR${}_{\mathrm{RC}}$(1)	$\hat{\alpha }=0.547$	$\hat{\theta }=2.0495$	494.906	506.7852
	$\hat{p}=0.185$	$\hat{\beta }=0.5188$		
